# Supplementary figures and images for: Live applications of norbormide-based fluorescent probes in Drosophila melanogaster
Source: PLoS One. 2019 Apr 8;14(4):e0211169. doi: 10.1371/journal.pone.0211169 (PMC6453474; doi:10.1371/journal.pone.0211169)

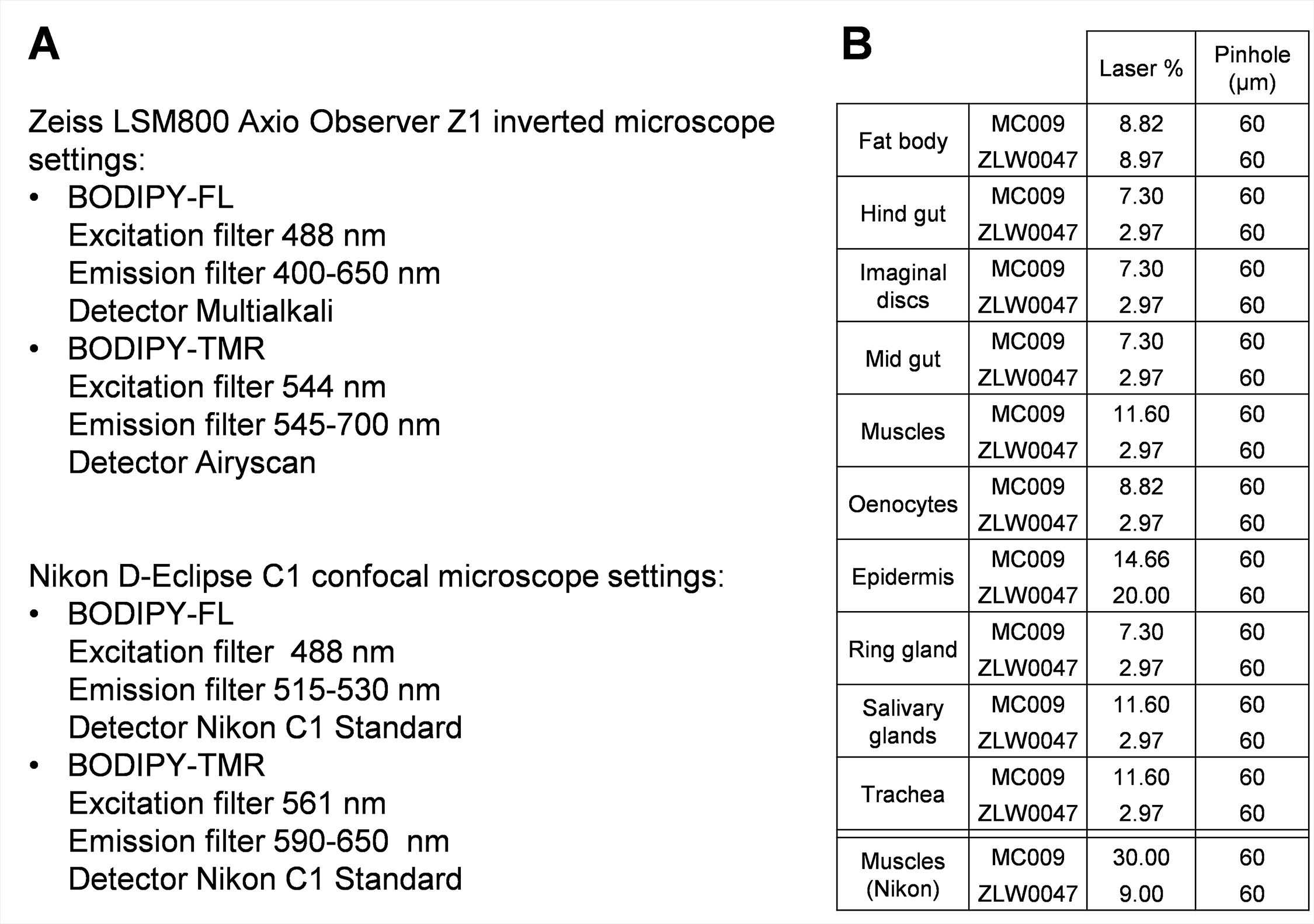

Supplement: S1 Fig — Zeiss LSM800 Axio Observer Z1 inverted microscope and Nikon D-Eclipse C1 confocal microscope settings used (A). Summary table of laser intensity (%) and pinhole size (μm) used to capture NRBMC009 and NRBZLW0047 in each tissue considered in the study (B). (TIF) [file pone.0211169.s001.tif]

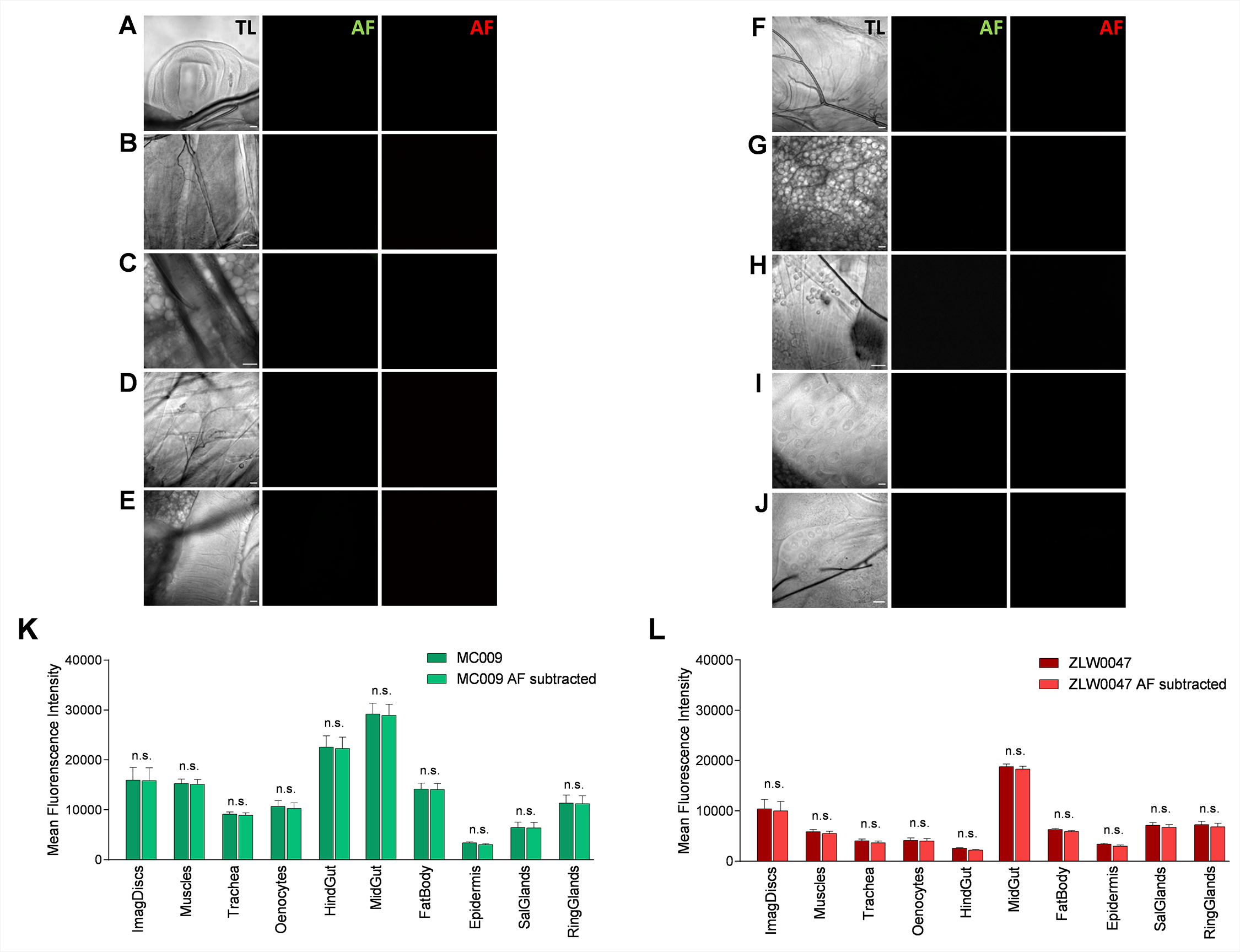

Supplement: S2 Fig — Confocal live cell images of unlabeled (A) leg imaginal disc, (B) muscles, (C) trachea, (D) oenocytes, (E) hindgut, (F) midgut, (G) fat body, (H) epidermis, (I) salivary gland, and (J) ring gland; magnification 40x; scale bars 20 μm. TL: transmitted light; green AF: autofluorescence using BODIPY-FL laser settings; red AF: autofluorescence using BODIPY-TMR laser settings. Quantification of NRBMC009 (K) and NRBZLW0047 (L) fluorescence intensity without (dark bars) or with (light bars) autofluorescence subtraction in different larval tissues. Data are expressed as mean ± SEM, n≥5; significance was calculated using unpaired t test; n.s. p > 0.05. (TIF) [file pone.0211169.s002.tif]

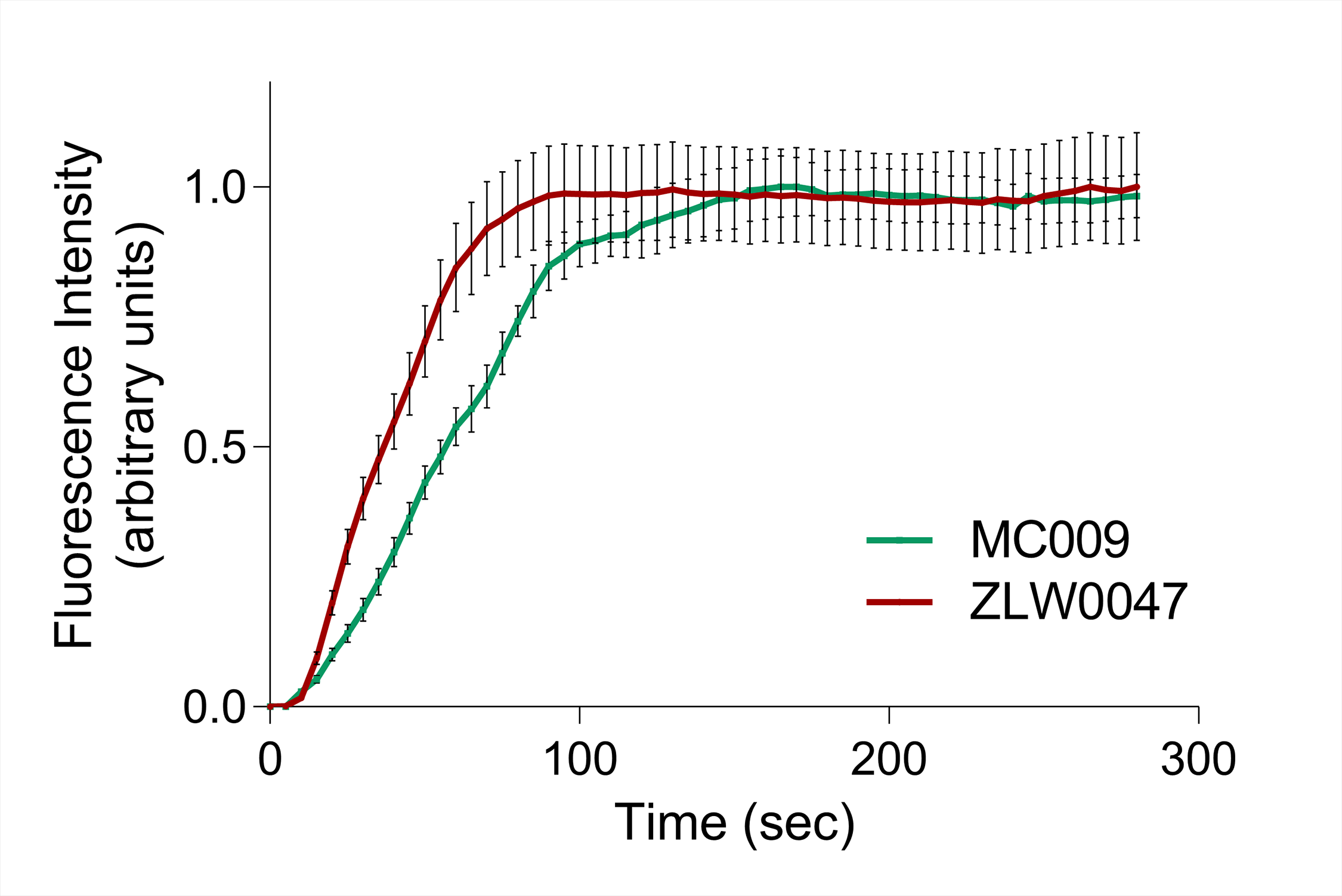

Supplement: S3 Fig — Fluorescence intensity of NRBMC009 and NRBZLW0047 internalization in dissected w[1118] larval muscles, extrapolated from a time-lapse image taken every 5 seconds for about 5 minutes. Data are expressed as mean ± SEM, n≥10. (TIF) [file pone.0211169.s003.tif]

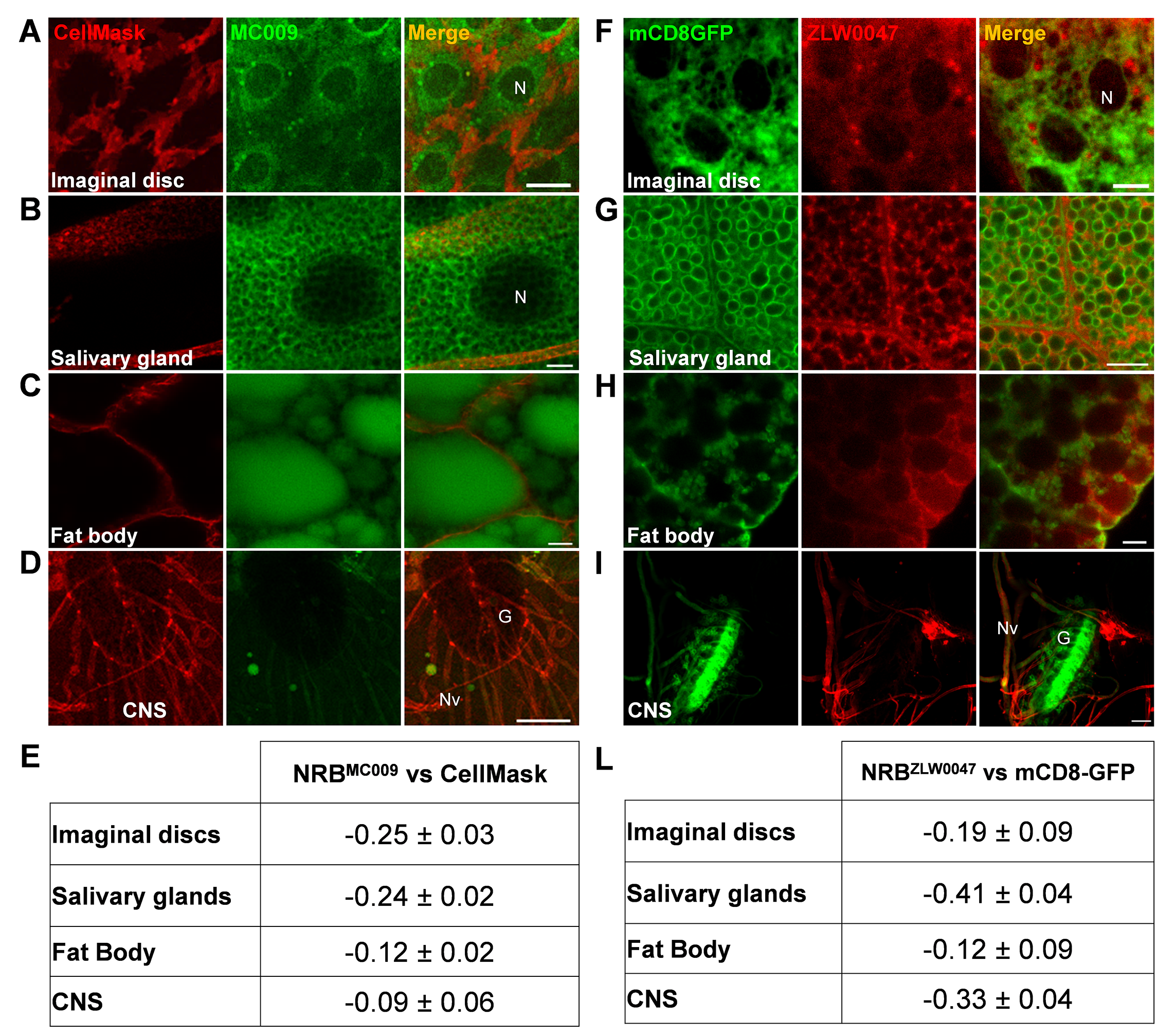

Supplement: S4 Fig — Confocal live imaging of w[1118] larval (A) peripodal membrane cells of a leg imaginal disc, (B) salivary gland, (C) fat body, and (D) central nervous system labeled with NRBMC009 500 nM (green) and CellMask Orange (cell membrane marker) 1 μM (red). Magnification 60x, scale bars 10 μm (A-C); magnification 40x, scale bar 100 μm (D). N: nucleus, G: ganglion, Nv: nerves. Summary table of Pearson’s correlation coefficients between NRBMC009 and CellMask in the evaluated tissues (E). Data are expressed as mean ± SEM, n≥10. Confocal live imaging of UAS-mCD8-GFP/Tubulin-Gal4 (cell membrane marker) larval (F) peripodal membrane cells of a leg imaginal disc, (G) salivary gland, (H) fat body, and (I) central nervous system labeled with NRBZLW0047 1 μM (red). Magnification 60x, scale bars 10 μm (A-C); magnification 40x, scale bar 100 μm (D). N: nucleus, G: ganglion, Nv: nerves. Summary table of Pearson’s correlation coefficients between NRBZLW0047 and mCD8-GFP signal in the evaluated tissues (L). Data are expressed as mean ± SEM, n≥10. (TIF) [file pone.0211169.s004.tif]
